# Supplementary material for: From sequence to enzyme mechanism using multi-label machine learning
Source: BMC Bioinformatics. 2014 May 19;15:150. doi: 10.1186/1471-2105-15-150 (PMC4229970; doi:10.1186/1471-2105-15-150)
Supplement: Additional file 2 — Java code of ml2db. Additional file ml2db_code.tar.gz contains the Java source code to run the multi-label machine learning experiments and save the results to database. The code’s Javadoc is included. [file 1471-2105-15-150-S2.zip › additional file 2/ml2db/ml2db/doc/test/ml2db/dataharness/LoadFirstValidArff.html]

LoadFirstValidArff


JavaScript is disabled on your browser.


- Overview
- Package
- Class
- Use
- Tree
- Deprecated
- Index
- Help

- Prev Class
- Next Class

- Frames
- No Frames

- All Classes

- Summary:
- Nested |
- Field |
- Constr |
- Method

- Detail:
- Field |
- Constr |
- Method


test.ml2db.dataharness

## Class LoadFirstValidArff

- java.lang.Object
- - test.ml2db.dataharness.LoadFirstValidArff

- ---

    

  ```
  public class LoadFirstValidArff
  extends java.lang.Object
  ```

  Class

  Version:
  :   7 Jul 2011

  Author:
  :   Luna De Ferrari ldf(at)st-andrews.ac.uk

- - ### Constructor Summary

    Constructors

    Constructor and Description

    `LoadFirstValidArff()`
  - ### Method Summary

    Methods

    | Modifier and Type | Method and Description |
    | --- | --- |
    | `static int` | `getFirstTestArffFromDb()` |
    | `static mulan.data.MultiLabelInstances` | `getFirstTestArffInstancesFromDb()` |
    | `static int` | `getFirstTrainArffFromDb()` |
    | `static mulan.data.MultiLabelInstances` | `getFirstTrainArffInstancesFromDb()` |
    | `static java.util.Vector<java.lang.String>` | `getFirstTrainPathFromDb()` Get the arff path and xml path |
    | `static java.lang.String` | `getTrainsetString()` |

    - ### Methods inherited from class java.lang.Object

      `equals, getClass, hashCode, notify, notifyAll, toString, wait, wait, wait`

- - ### Constructor Detail


    - #### LoadFirstValidArff

      ```
      public LoadFirstValidArff()
      ```
  - ### Method Detail


    - #### getFirstTestArffFromDb

      ```
      public static int getFirstTestArffFromDb()
      ```


    - #### getFirstTestArffInstancesFromDb

      ```
      public static mulan.data.MultiLabelInstances getFirstTestArffInstancesFromDb()
      ```


    - #### getFirstTrainArffFromDb

      ```
      public static int getFirstTrainArffFromDb()
      ```


    - #### getFirstTrainArffInstancesFromDb

      ```
      public static mulan.data.MultiLabelInstances getFirstTrainArffInstancesFromDb()
      ```


    - #### getFirstTrainPathFromDb

      ```
      public static java.util.Vector<java.lang.String> getFirstTrainPathFromDb()
      ```

      Get the arff path and xml path

      Returns:
      :   the arff path (index 0) and xml path (index 1)


    - #### getTrainsetString

      ```
      public static java.lang.String getTrainsetString()
      ```


- Overview
- Package
- Class
- Use
- Tree
- Deprecated
- Index
- Help

- Prev Class
- Next Class

- Frames
- No Frames

- All Classes

- Summary:
- Nested |
- Field |
- Constr |
- Method

- Detail:
- Field |
- Constr |
- Method
